# Strawberry Polyphenols Attenuate Ethanol-Induced Gastric Lesions in Rats by Activation of Antioxidant Enzymes and Attenuation of MDA Increase

**DOI:** 10.1371/journal.pone.0025878

**Published:** 2011-10-07

**Authors:** José M. Alvarez-Suarez, Dragana Dekanski, Slavica Ristić, Nevena V. Radonjić, Nataša D. Petronijević, Francesca Giampieri, Paola Astolfi, Ana M. González-Paramás, Celestino Santos-Buelga, Sara Tulipani, José L. Quiles, Bruno Mezzetti, Maurizio Battino

**Affiliations:** 1 Dipartimento di Scienze Cliniche Specialistiche ed Odontostomatologiche – Sez. Biochimica, Facoltà di Medicina, Università Politecnica delle Marche, Ancona, Italy; 2 Biomedical Research, R&D Institute, Galenika a.d., Belgrade, Serbia; 3 Institute of Medical and Clinical Biochemistry, School of Medicine, University of Belgrade, Belgrade, Serbia; 4 ISAC-Chemistry Division, Marche Polytechnic University, Ancona, Italy; 5 Grupo de Investigación en Polifenoles (GIP-USAL), Faculty of Pharmacy, Salamanca University, Campus Miguel de Unamuno, Salamanca, Spain; 6 Department of Nutrition and Food Science-CeRTA, Faculty of Pharmacy, University of Barcelona, Barcelona, Spain; 7 Department of Physiology, Institute of Nutrition and Food Technology “José Mataix”, Biomedical Research Center, University of Granada, Granada, Spain; 8 Department of Environmental and Crop Science (SAPROV), Faculty of Agriculture, Marche Polytechnic University, Ancona, Italy; Enzo Life Sciences, Inc., United States of America

## Abstract

**Background and Aim:**

Free radicals are implicated in the aetiology of gastrointestinal disorders such as gastric ulcer, colorectal cancer and inflammatory bowel disease. Strawberries are common and important fruit due to their high content of essential nutrient and beneficial phytochemicals which seem to have relevant biological activity on human health. In the present study we investigated the antioxidant and protective effects of three strawberry extracts against ethanol-induced gastric mucosa damage in an experimental *in vivo* model and to test whether strawberry extracts affect antioxidant enzyme activities in gastric mucosa.

**Methods/Principal Findings:**

Strawberry extracts were obtained from Adria, Sveva and Alba cultivars. Total antioxidant capacity and radical scavenging capacity were performed by TEAC, ORAC and electron paramagnetic resonance assays. Identification and quantification of anthocyanins was carried out by HPLC-DAD-MS analyses. Different groups of animals received 40 mg/day/kg body weight of strawberry crude extracts for 10 days. Gastric damage was induced by ethanol. The ulcer index was calculated together with the determination of catalase and SOD activities and MDA contents. Strawberry extracts are rich in anthocyanins and present important antioxidant capacity. Ethanol caused severe gastric damage and strawberry consumption protected against its deleterious role. Antioxidant enzyme activities increased significantly after strawberry extract intake and a concomitantly decrease in gastric lipid peroxidation was found. A significant correlation between total anthocyanin content and percent of inhibition of ulcer index was also found.

**Conclusions:**

Strawberry extracts prevented exogenous ethanol-induced damage to rats' gastric mucosa. These effects seem to be associated with the antioxidant activity and phenolic content in the extract as well as with the capacity of promoting the action of antioxidant enzymes. A diet rich in strawberries might exert a beneficial effect in the prevention of gastric diseases related to generation of reactive oxygen species.

## Introduction

Gastric epithelium is often attacked by physical, chemical or microbiological agents acting from the gastric lumen. Among the numerous injurious luminal agents and irritants of both exogenous and endogenous origin, the stomach is a site of massive production and concentration of reactive oxygen species (ROS), far higher than other tissues or biological fluids [1]. The generation of ROS plays a major role in the development of multiple pathologies, such as gastritis, peptic ulcerations or gastric adenocarcinoma [2]. Gastric mucosal layers thus represent a dynamic barrier in counteracting the effects of noxious agents through a series of endogenous antioxidant defense systems.

Also diet may exert multiple protective biological effects on the mucosa of the gastrointestinal tract. Fruits and vegetables, in fact, seem to play a preventive role against the development of gastric erosions, ulcerations and cancer. This action, called gastro- or cyto-protection, does not imply the inhibition of gastric acid secretion and must be taken into account as a potential tool for gastroprotection against the action of various irritants and ulcerogens [1], [3].

In the last decade, a great deal of interest has been particularly addressed to phenolic compounds, among the major class of phytochemical antioxidants in fruits and vegetables. The *in vitro* capacity of polyphenols to act as both primary and secondary antioxidants has been probably the best described property of almost every group of flavonoid and non-flavonoid compounds. This concept, however, appears now to be an oversimplified view of their mode of action [4]. Emerging findings, in fact, suggest a variety of other potential mechanisms of action of polyphenols in cytoprotection against oxidative stress, even at the pharmacological doses that they reach *in vivo*, which may also be independent from conventional direct antioxidant-reducing activities.

The strawberry (*Fragaria×ananassa*, Dutch) is one of the most commonly consumed berries worldwide. The high variety and content of polyphenolic constituents of strawberries, mainly represented by flavonoid anthocyanins and non-flavonoid condensed tannins (ellagitannins), have recently attracted a growing attention [5]–[9]. Relatively few studies concerning the *in vivo* antioxidative effects and behavior of anthocyanins after ingestion have been reported [10]–[12]. However, evidence already support the hypothesis that anthocyanins exhibit their cytoprotective activities *via* a multitude of biochemical mechanisms [13], and that they seem to offer an indirect antioxidant protection by activating cellular antioxidant enzymes, which are crucial components of the antioxidant defense system in the body.

For many years our group has focused its research objectives on the characterization of nutritional quality of several strawberry genotypes and on evaluating the effect of acute and protracted strawberry consumption on the antioxidant status in human subjects [14]–[18]. In particular, the genetic background plays an important role on the nutritional quality of strawberry fruits [19], [20], since the content of micronutrients and phytochemicals may greatly vary from cultivar to cultivar. However, few genotypes have been well characterized for these important features [21], and data are now available on the possibility of improving strawberry nutritional traits by breeding processes for their possible beneficial effects in human health. Interestingly, we found that the consumption of strawberries seems to increase both plasma and cellular antioxidant defenses [14]–[18].

Based on the results of previous studies, we asked ourselves whether strawberry extracts were able to directly protect gastric mucosa against damages mediated by noxious agents, such as ethanol, through the possible interaction between their phytochemical antioxidant compounds and endogenous antioxidant systems in rats. Therefore, this study intended to evaluate the *in vivo* effects of three different strawberry extracts (Alba, Adria and Sveva) which had been previously well-characterized and selected for having given the best results as for as nutritional quality is concerned [14]–[19], [21]. The strawberry extracts were challenged in an experimental model of ethanol-induced gastric mucosal damage in rats, and to test whether they could affect antioxidant enzyme activity in gastric mucosa.

## Materials and Methods

### Chemicals

All chemicals and solvents were of analytical grade. ABTS (2,2′-azino-bis-(3-ethylbenzothiazolne-6-sulfonic acid) diammonium salt), Trolox (6-hydroxy-2,5,7,8-tetramethyl-chroman-2-carboxylic acid), bovine serum albumin (BSA) and fluorescein were purchased from Fluka Chemie (Buchs, Switzerland). AAPH (2,2′-azobis(2-methylpropionamidine) dihydrochloride), butylated hydroxytoluene (BHT), thiobarbituric acid (TBA), hydrogen peroxide (H_2_O_2_), quercetin and polyethylene glycol 400 (PEG-400) were purchased from Sigma-Aldrich Chemie GmbH (Steinheim, Germany). Cyanidin 3-O-β-glucopyranoside and Pelargonidin 3-O-β-glucopyranoside were purchased from Polyphenols Laboratories AS, Norway. Xanthine oxidase (EC 1.1.3.22) was from Serva Electrophoresis GmbH. 5,5-Dimethyl-1-pyrroline-N-oxide (DMPO) was purchased from Enzo Life Science AG (Lausen, Switzerland) and was used without further purification (no residual signals were detected in DMPO water solutions).

### Animals

Male Wistar rats (*Rattus norvegicus*) from Biomedical Research Center, R&D Institute, Galenika a.d. (Belgrade, Serbia), weighing 180–220 g, were used in this study. Rats were housed 3 per cage under constant environmental conditions (20–24°C; 12-h light–dark cycle) and were given *ad libitum* access to standard pelleted food and water. All animals were fed with control diet for 7 days before the start of the experiment as a conditioning period. The study was approved [permit number 486/2 (2008)] by the Committee on the Ethics of Animal Experiments, Medical School, University of Belgrade, and was carried out in strict accordance with the statements of the European Union regarding the handling of experimental animals (86/609/EEC).

### Strawberry material

Ripe strawberry fruits from three selected cultivars (Alba, Adria and Sveva), were harvested at the experimental field for genetic improvement of the Azienda Agraria Didattico Sperimentale (Marche Polytechnic University, Ancona, Italy). Within 2 h after harvest, whole fruits were stored at −20°C for further analysis.

### Sample preparation

For the analysis of fruit antioxidant capacity, a hydroalcoholic extract was obtained as previously described [19]. Briefly, compounds were extracted by homogenizing (Ultraturrax T25, Janke & Kunkel, IKA Labortechnik) for 2 min 100 g of fruit samples in 1 L of extraction solution, consisting of methanol and Milli-Q water (80% v/v) and stirring for 2 h in the dark at room temperature. The mixture was centrifuged in two sequential times for 15 min at 1200*×*g, and supernatant was filtered through a 0.45 µm Minisart filter (PBI International) before analysis. Moreover, for the evaluation of gastric lesions (see below), hydroalcoholic phases were combined, water was added and the supernatant was concentrated under vacuum in a rotary evaporator at <30°C up to total evaporation of the methanol, giving ∼7.5 gram of semi-liquid extract, subsequently used in the analysis of induction and evaluation of gastric lesions.

Anthocyanin extraction for HPLC-MS analysis was performed as previously described [8]. Frozen strawberries (50 g) were homogenized in methanol containing 0.1% HCl, kept overnight (∼14 h) at 3–5°C and later filtered through a Büchner funnel under vacuum. The solid residue was exhaustively washed with methanol; the filtrates obtained were centrifuged (4000*×*g, 15 min, 21°C) and the solid residue further submitted to the same process for the number of times necessary to complete color extraction. The aqueous extract obtained was washed with *n*-hexane to remove liposoluble substances and then an aliquot (2 ml) of the aqueous phase was carefully deposited onto a C-18 SepPaks Vac 6 cc cartridge (Waters). Sugars and more polar substances were removed by passing 15 ml of ultrapure water and anthocyanin pigments further eluted with 5 ml of methanol: 0.1% trifluoroacetic acid (95∶5). The methanolic extract was concentrated under vacuum in a rotary evaporator at <30°C, after adding water. The aqueous extract was collected, its volume completed to 2 ml with ultrapure water and filtered through a Minisart filter of 45 µm (PBI International, Milan, Italy) for HPLC analysis. For each strawberry variety, three independent extracts were prepared, purified and analyzed separately.

### Quantification of the total antioxidant capacity (TAC)

The TAC of the hydroalcoholic extracts was determined using in parallel the Trolox equivalent antioxidant capacity (TEAC) and the oxygen radical absorbance capacity (ORAC) assays.

The TEAC assay was carried out according to the recently modified method of Re and co-workers [22] and combined to a flow injection analysis (FIA) system as previously set up by our group [23]. This TEAC method, also called the FIA-ABTS decolorization assay, is based on the ability of antioxidant compounds to quench the ABTS radical cation (ABTS^+^) and reduce the radical to the colorless neutral form. The undiluted strawberry extract (10 µL) is injected into a serpentine-knotted reaction coil and allowed to react with the ABTS^+^ working solution pumped into the coil at a flow rate of 1.2 mL/min. The extent of decolorization, expressed as percentage of inhibition of absorbance, is then plotted as a function of concentrations of the antioxidants in the sample. TEAC results are expressed as micromoles of Trolox equivalents per gram of fresh weight (FW) of strawberry (µmol of TE/g of FW). Data are reported as a mean value ± SD for four measurements.

The ORAC assay was based on the procedure previously described [24]. Free radicals are produced by the radical generator AAPH which oxidize the fluorescent compound fluorescein leading to loss in fluorescence. All reagents are prepared in phosphate buffer (pH 7.0, 75 mM) and Trolox (25–150 µmol) is used as standard. The hydroalcoholic extracts are suitably diluted in the phosphate buffer. Each well of a 96 well microplate contains, in a final volume of 200 µL assay solution, 150 µL of fluorescein (0.08 µM) and 25 µL of the undiluted strawberry extract, preincubated for 10 min at 37°C, then 25 µL of AAPH (150 mM) are added. After addition of AAPH, the plate is shaken automatically for 3 seconds and the fluorescence is measured every 2 min for 120 min with emission and excitation wavelengths of 530 and 485 nm, respectively, using a microplate fluorescence reader (SynergyTM Multi-Detection Microplate Reader; Bio-Tek®, Instruments, Inc., USA) that is maintained at 37°C. The ORAC values are calculated as area under the curve and expressed as micromole of Trolox equivalent per gram of FW of strawberry (µmol TE/g of FW).

### Detection of hydroxyl radical scavenging capacity measured by Electron Paramagnetic Resonance (EPR-spin trapping)

EPR spectroscopy, in combination with the spin-trapping technique, was used to investigate the hydroxyl radical (OH^•^) scavenging activity. The OH^•^ scavenging capability of strawberry extracts was examined by its addition to the OH^•^-generating Fenton system following the procedure previously described [25]. Briefly, 150 µL of 0.1 M DMPO (in water) were added to 38 µL of appropriated diluted strawberry extract (1∶100 v/v) in an eppendorf tube and gently mixed by pipetting. To this reaction solution, 2 µL of 0.06 M H_2_O_2_ were added followed by 5 µL of freshly prepared and nitrogen-flushed 0.5 mM FeSO_4_ to initiate the reaction. The reaction mixture was immediately transferred into a sample tube and measurements taken 60 s after FeSO_4_ addition. EPR spectra were recorded on a Bruker EMX EPR spectrometer (Bruker, Karlsruhe, Germany) operating at X-Band equipped with an XL microwave frequency counter, with the following settings: frequency 9.78 GHz, power 25 mW, modulation amplitude 0.5 G (Gauss), gain 5×105, field width 100 G, time constant 0.64 ms, scan time 21 s. As a control, the EPR spectrum obtained from a reaction mixture containing DMPO, H_2_O_2_ and FeSO_4_ (positive control) was used and the corresponding signal amplitude was compared to those of the DMPO-OH adducts recorded in the presence of strawberry. The decrease in the signal amplitude in the test samples correlates with their radical scavenging activity.

In order to check for the absence of radical species in the sample, a negative control was prepared by simply mixing the DMPO and strawberry extract. The decrease in the signal amplitude in the test samples correlates with their radical scavenging activity. The height of the second peak was recorded and the % radical scavenging capacity (%RSC) was then calculated according to equation:
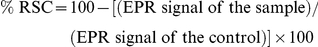
The results are expressed as the concentration of strawberry extract required to inhibit the rate of OH^•^ radical generation by 50% (IC_50_) (mg/ml).

### Identification and quantification of red pigments in strawberry fruit by HPLC-DAD-MS analysis

Analyses were performed in a Hewlett-Packard 1100 series liquid chromatograph. Separation was achieved on a 5 µm AQUA® C 18 150 mm×4.6 mm column (Phenomenex, Torrance, CA) thermostated at 35°C. Solvents used were: (A) 0.1% trifluoroacetic acid in water, and (B) HPLC-grade acetonitrile, establishing the following gradient: isocratic 10% B for 5 min, 10–15% B over 15 min, isocratic 15% B for 5 min, 15–18% B over 5 min, and 18–35% B over 20 min, using a flow rate of 0.5 ml/min. Double on-line detection was carried out in a diode array detector (DAD), using 520 nm as the selected wavelength, and a mass spectrometer (MS) connected to the HPLC system via the DAD cell outlet. The mass spectrometer was a Finnigan LCQ (San Jose, CA) equipped with an ESI source and an ion trap mass analyzer, which was controlled by the LCQ Xcalibur software. Nitrogen was used as both auxiliary and sheath gas at flow rates of 6 and 1.2 L/min, respectively. The capillary voltage was 4 V and the capillary temperature 195°C. Spectra were recorded in positive ion mode between *m/z* 150 and 1500. The MS detector was programmed to perform a series of three consecutive scans: a full scan, a zoom scan of the most abundant ion in the first scan and an MS–MS scan of the most abundant ion, using a normalized collision energy of 45%.

Anthocyanins in the strawberry were quantified from the areas of chromatographic peaks recorded at 520 nm by comparison with calibration curves obtained with external standards of Cyanidin-3-glucoside (for cyanidin-based anthocyanins) and of Pelargonidin 3-glucoside (for pelargonidin-based anthocyanins). Strawberry extracts were analysed in triplicate.

### Induction of gastric lesions

Before the experiment, the animals were randomly divided into 6 groups (6 rats in each group). Control group consisted of healthy animals with no pretreatment and no ethanol-induced lesions, ethanol group received polyethylene glycol 400 (PEG 400) for 10 days and then 1 ml of ethanol; positive control group received 100 mg/kg of body weight of quercetin dissolved in 10% PEG 400 for 10 days. As far as the groups treated with the three different cultivars are concerned, the rats daily received 40 mg/kg body weight of strawberry crude extract dissolved in 10% PEG 400 for 10 days. The administration of extracts was carried out intragastrically (i.g.) using a gavage. The dose was calculated according to the weight of fresh fruit that corresponds to the daily consumption of 500 grams of strawberries for a human healthy adult of 70 kg, as previously reported by our group [14]. On the day before the induction of gastric lesions, rats were placed in individual metabolic cages and deprived of food, with free access to tap water for 18–20 hours. The last administration of strawberry extract was 120 min before absolute ethanol administration that induced gastric lesions.

One hour after i.g. administration of 1 ml of ethanol, rats were sacrificed by cervical dislocation; the stomach was removed and opened along the greater curvature. The stomach content was drained and completely recovered by washing with 10 ml of isotonic saline. The content and washing were combined and centrifuged (3500*×*g, 10 min.) and the pH value of supernatant was measured. The stomach was rinsed with water, pinned open for macroscopic examination and for photo-documentation by a digital camera (SONY DSC-H50, Japan) and stored at −80°C until further analysis.

Areas of gastric hemorrhagic lesions were measured and calculated as previously described [26] by planimetry using the Image Processing and Analysis in Java (ImageJ) by the National Institutes of Health (NIH, USA); ulcer index (UI) and percent of inhibition of UI in relation to the control group were estimated using the following equations:
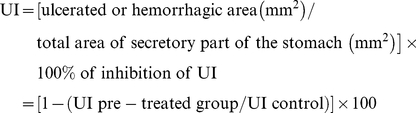



### Biochemical evaluation of gastric mucosa

Mucosal tissue from each animal was scraped from the stomach with a blunt knife and the tissue was weighed, transferred to the ice-cooled test tube and homogenized by Ultra-Turrax T25, (Janke & Kunkel Gmbh & Co., IKA®-Labortechnik, Staufen, Germany) in 20 mmol/l Tris buffer, pH 7.4, containing 5 mmol butylated hydroxytoluene (BHT) to prevent new lipid peroxidation that can occur during homogenization. The homogenate was then centrifuged at 12000*×*g at 4°C, (Megafuge 2.0.R, Heraeus, Germany) for 10 min. Supernatant was aliquoted and stored at −80°C until determination of total protein content, catalase (Cat), superoxide dismutase (SOD) and malondialdehyde (MDA).

The protein content of tissue samples was estimated by the method of Lowry [27] using bovine serum albumin (BSA) as standard.

MDA formation was determined as a measure of lipid peroxidation using a modified TBA assay [28] determined spectrophotometrically (UV-vis spectrophotometer HP 8453, Agilent Technologies, Santa Clara, C.A.) at 533 nm.

Activity of Cat in gastric mucosa was determined according to the procedure of Goth [29] by following the absorbance of hydrogen peroxide at 230 nm and pH 7.0.

SOD activity in the gastric mucosa was determined by measuring the inhibition of autoxidation of adrenaline at pH 10.2 at 30°C using the Misra and Fridovich method [30]. One unit of SOD activity represents the amount of SOD necessary to cause 50% inhibition of adrenaline auto-oxidation.

### Statistical analysis

Shapiro-Wilk normality test was used to compare distributions. One-way ANOVA followed by Turkey's Post Hoc Test was performed with the OriginPro 7.5. The IC_50_ was calculated by nonlinear regression analysis using the equation for a sigmoid concentration–response curve (GraphPad Prism). All data are presented as means ± SD; *p*≤0.05 was considered as significant.

## Results

### Total Antioxidant Capacity

The TAC of whole fruit extracts was quantified by the ORAC and TEAC assays (Table 1). Cultivars Adria and Sveva showed the highest TAC values (averaging 51.59 and 51.09 µmol of TE/g of FW, respectively) according to the ORAC assay; no significant difference was found between them, whereas the lowest TAC was found in Alba (34.76 µmol of TE/g of FW, ORAC value), which differs significantly (*p<0.05*) from the rest of the cultivars studied. Moreover, the TEAC results showed that Sveva presents the highest TAC values (25.16 µmol of TE/g of FW), whereas the lowest TAC was found in Alba (13.25 µmol of TE/g of FW); a significant difference (*p<0.05*) was found among all cultivars. A correlation was found between ORAC and TEAC assay (r = 0.75, *p≤0.05*). TEAC values were in each case slightly lower than the corresponding ORAC values and this is most likely due to the intrinsic differences between the two assays. In fact, the TEAC method measures the capacity of the sample to reduce only one type of radical, the ABTS^+^; whilst the ORAC assay measures the capacity of the sample to scavenge a variety of radicals initially triggered by the carbon-centered radicals generated upon decomposition of AAPH. The latter rapidly react in the presence of oxygen to give peroxyl radicals, which can further react to give alkoxy radicals that may interfere with the oxidation of fluorescein and with the sample.

**Table 1:** Total antioxidant capacities of the strawberry cultivar on study.

Cultivar	ORAC(µmol/g FW)	TEAC(µmol/g FW)	OH^•^ Radical Scavenging Capacity(IC_50_ mg/ml)
**Sveva**	51.03±3.65^a^	25.16±0.75^a^	0.51^a^
**Adria**	51.59±7.14^a^	17.49±0.83^b^	0.46^b^
**Alba**	34.76±3.08^b^	13.25±3.96^c^	0.49^a^

Data are means ± standard deviation. Mean values within a column sharing the same letter are not significantly different by Tukey's multiple range test (*p<0.05*).

### EPR assay

**Figure 1:**
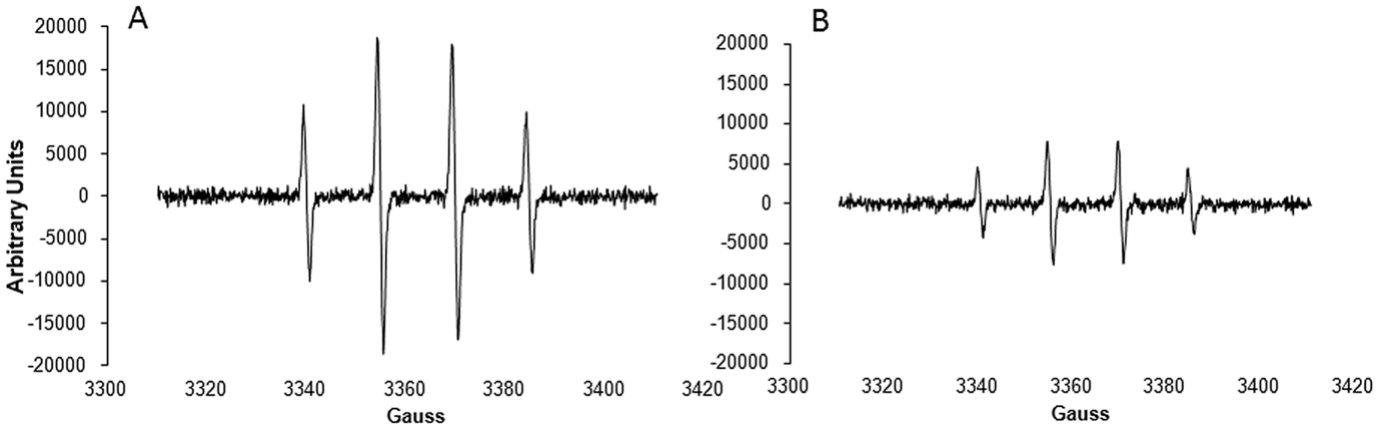
Hydroxyl radical scavenging activities of strawberry extracts. Positive control (DMPO, H_2_O_2_ and FeSO_4_) signal amplitude was compared to those of the DMPO-OH adducts recorded in the presence of strawberry extracts. The decrease in the signal amplitude in the samples correlates with their radical scavenging activity. (A) Typical EPR spectrum of the OH^•^ radical trapped with DMPO from the Fenton reaction (positive control), (B) typical EPR spectra obtained after the strawberry extract was added.

The OH^•^ radical was formed from hydrogen peroxide by an Fe(II)-catalyzed Fenton reaction and determined by the EPR-spin trapping technique. As shown in Figure 1A, the reaction of Fe^2+^ and H_2_O_2_ in the presence of the spin trapping agent DMPO, generated the ‘spin-adduct’ DMPO-OH, characterized by a four line spectrum with a 1∶2∶2∶1 quartet of lines in the EPR spectrum with the hyperfine coupling parameters (a_N_ = a_H_^β^ = 14.85 G). Strawberry extracts produced a concentration-dependent inhibition of the EPR signal intensity of DMPO-OH spin adduct. Figure 1B presents a typical EPR spectrum after the extracts were added, showing a significant (*p<0.05*) decrease in the DMPO-OH signal amplitude. Moreover, it was not found a significant difference between the extracts; the IC_50_ in the three strawberries extracts ranges between 0.51 mg/mL, 0.48 mg/mL and 0.46 mg/mL for Sveva, Adria and Alba respectively (Table 1).

### Pigment identification

**Figure 2:**
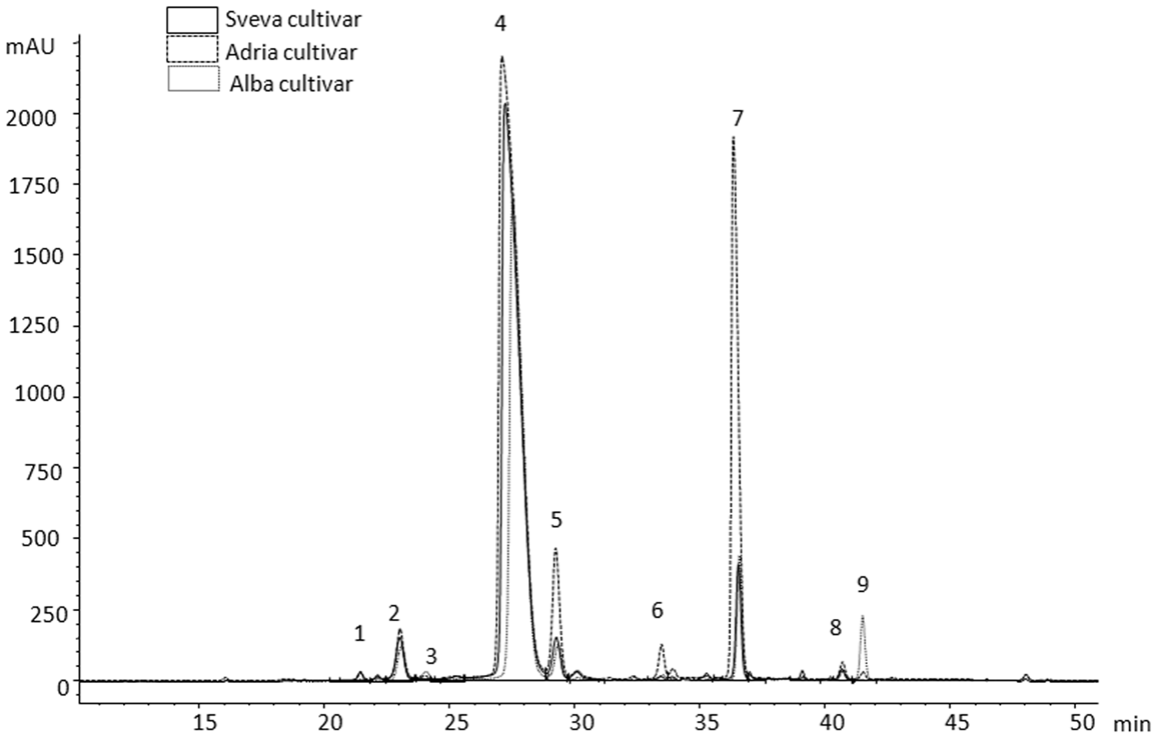
Representative HPLC-DAD chromatograms recorded at 520 nm showing the anthocyanin profiles of the strawberry samples. Adria, Alba and Sveva cultivars. Peaks: 1, (Epi)afzelechin-(4→8)Pg 3-glucoside; 2, Cy-3-glucoside; 3, Pg 3,5-diglucoside; 4, Pg 3-glucoside; 5, Pg 3-rutinoside; 6, Cy 3-malonylglucoside; 7, Pg 3-malonylglucoside; 8, Pg 3-acetylglucoside; 9, Pg 3-succinylglucoside or Pg 3-methylmalonylglucoside.

In the three different strawberry varieties analysed 9 anthocyanin pigments were detected. The compounds were identified on the basis of their UV-vis and mass spectra obtained by HPLC-DAD-ESI/MS in positive mode, as well as their chromatographic behavior compared to external standards when available. Figure 2 shows a representative HPLC chromatograms of anthocyanin profiles in the three strawberry cultivars analysed (i.e. Alba, Adria and Sveva). Peak data obtained in HPLC-DAD-MS analyses (retention time in the HPLC system, λ_max_ in the visible region, molecular ion and main fragments observed in MS^2^) are summarized in Table 2. In addition to the compounds indicated in the table, other very minor pigments were also detected, although no good absorption or mass spectra could be obtained to allow speculation about their identity. Thus, λ_max_ of the peaks of the Pg-based anthocyanins vary from 500 nm (peak 3) to 508 nm (peak 9). Peak 1 was found only in the Adria cultivar and was assigned to condensed pigments containing C–C linked anthocyanin (Pelargonidin, Pg) and flavanol (afzelechin) residues. This compound showed a molecular ion [M+H]^+^ at *m/z* 705, releasing major MS^2^ fragments at *m/z* 543, 407, 313 and 271. Its UV-vis, mass spectrum and retention time were consistent with the (Epi)afzelechin-(4→8)-Pg 3-glucoside.

**Table 2:** Retention time (R*_t_*), wavelengths of maximum absorption in the visible region (λ_max_), mass spectral data, tentative identification and total anthocyanin content in the three strawberry cultivars in study.

Peak	R_t_ (min)	λ_max_ (min)	Molecular ion		Tentative identification	Total anthocyanin content (mg/Kg of FW)		
			[M^+^] (*m/z*)	MS^2^ (*m/z*)		Sveva	Alba	Adria
1	21.7	515	705	543,407,	(Epi)afzelechin-(4→8)Pg 3-	nd	nd	2.73±0.1
				313,271	glucoside			
2	23.2	515	449	287	Cy-3-glucoside	16.35±5.6	19.02±3.5	28.97±4.6
3	24.1	500	595	433, 271	Pg 3,5-diglucoside	nd	nd	0.438±0.01
4	27.3	502	433	271	Pg 3-glucoside	611.18±25.5	507.51±25.4	1054.3±67.4
5	29.3	503	579	433, 271	Pg 3-rutinoside	nd	20.89±4.1	101.03±21.5
6	33.7	n.a	535	287	Cy 3-malonylglucoside	nd	1.17±0.01	nd
7	37.7	504	519	271	Pg 3-malonylglucoside	2.85±0.4	65.48±5.6	2.350±0.2
8	40	504	475	271	Pg 3-acetylglucoside	7.250±0.2	1.13±0.01	nd
9	41.8	508	533	271	Pg 3-succinylglucoside? or	nd	nd	3.83±0.03
					Pg 3-methylmalonylglucoside?			
**Total anthocyanin content**						637.63±65.8	615.2±53.8	1193.64±101.4

Values expressed as means (mg/kg of FW) ± SD. nd, not detected.

The presence of pelargonidin (Pg) as anthocyanin in those peaks was further confirmed by their mass spectra, which showed MS^2^ signal at *m/z* [M]^+^ 271. Up to five peaks could be assigned to Pg derivates (peaks 3, 4, 5, 7, 8). In addition, two peaks (2 and 6) were identified as cyanidin (Cy) derivatives, based on the presence of a signal at *m/z* [M]^+^ 287 in their MS^2^ spectra, while one peak remains tentatively identified as Pg-derivatives (peak 9), and found only in Adria cultivar. No further contribution to the identification of this compound could be made in our study and, thus, remains unidentified. Similar profiles of anthocyanin were found in all strawberry varieties studied, although they differed in quantitative contents, where Adria presented the highest total anthocyanin content (*p<0.05*) (1193 mg/Kg FW) compared with Sveva (637.6 mg/Kg FW) and Alba (615 mg/Kg FW). In all samples, the major peak in the HPLC chromatograms corresponded to Pg 3-glucoside (peak 4).

### Protective effect of strawberry extracts on gastric injury in rats

**Figure 3:**
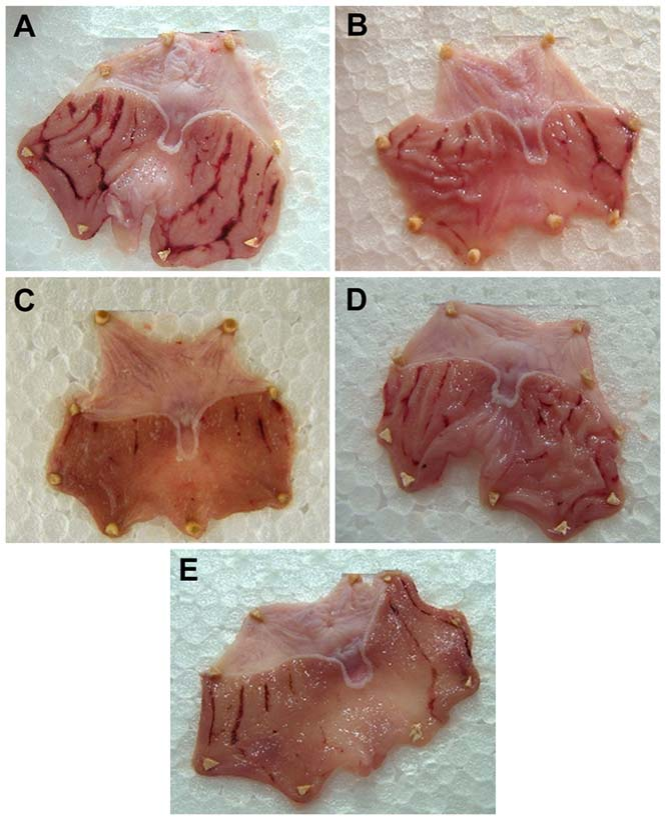
Protective effect of strawberry extracts on gastric injury in rats. Representative rat stomachs exposed to absolute ethanol for one hour: (A) non-pretreated rats, (B) rats pretreated with 100 mg/kg of Quercetin. Rats pretreated with 40 mg/kg of strawberry crude extracts once per day for 10 days: (C) Adria extract, (D) Alba extract and (E) Sveva extract. The last administration of strawberry extract was 2 h before absolute ethanol administration that induced gastric lesions.

In this study, gastric mucosal injury was induced by administration of ethanol through intubation into rat stomachs. After opening, gastric lesions were found in mucosa and consisted of elongated bands, 1–10 mm long, usually parallel to the long axis of the stomach. They were located mostly in the corpus, the portion of stomach secreting acid and pepsin. No visible lesions developed in the non-secretory part of the stomach (Fig. 3A).

**Figure 4:**
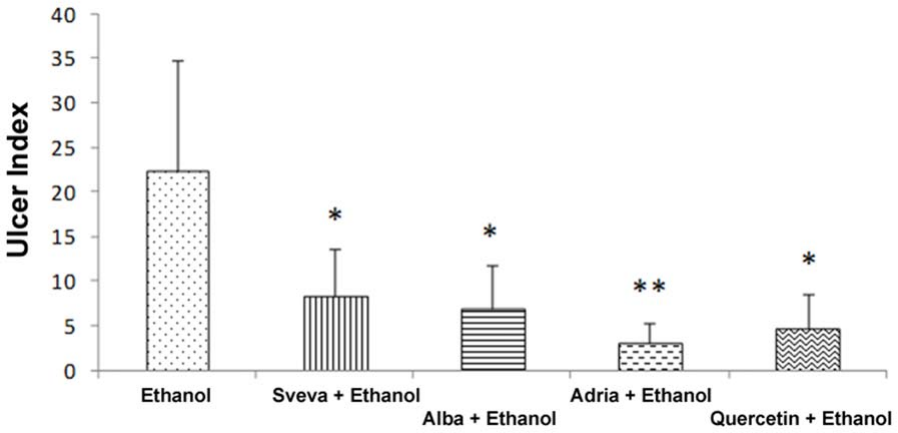
Effect of intragastric pretreatment with the different strawberry extract and quercetin on the ulcer index induced by ethanol. ^*^ Significant inhibition as compared to the value in the ethanol group (*p<0.05*).

Quercetin (Fig. 3B) and the three strawberry extracts (Fig. 3C for Adria, D for Alba and E for Sveva) showed significant protective effect on gastric mucosal injury, when they were given to rats before the administration of ethanol. Figure 4 shows the effects on the ulcer index of pretreatment with the three strawberry extracts and quercetin, as positive control, applied intragastrically. Ethanol caused typical widespread gastric lesion covering 22.3% of total stomach area. Pretreatment with 40 mg/kg body weight of the strawberry crude extracts significantly reduced (*p<0.001*) gastric lesions induced by absolute ethanol. Adria strawberry extracts presented the same protective effects as quercetin in suppressing the area of gastric mucosal damage. A significant difference (*p<0.05*) was found between Adria and the rest of the cultivars studied; in particular, in the group of animals pretreated with this cultivar, ethanol caused gastric lesions in only 3.01% of the total stomach area, representing an 87% of UI inhibition, related to the control group. These effects were similar to those obtained with quercetin, which showed a 79% UI inhibition compared to the ethanol group. A significant correlation between total anthocyanin content and % of UI inhibition was found (r = 0.9756, *p≤0.05*).

Moreover, it was also found that the red pigment fraction did not inhibit gastric acid secretion (Table 3), because no significant difference was found between the intragastric pH of pretreated rats and controls.

**Table 3:** Effect of strawberry extract given orally on gastric acid secretion in rats^a^.

Sample	pH
Control	5.61±1.03
Sveva cultivar	5.23±1.58
Alba cultivar	5.11±1.66
Adria cultivar	4.66±1.61
Quercetin 100 mg/kg	5.03±1.40

*^a^*no significant difference (*p<0.05*) was found between the intragastric pH of rats pretreated and controls.

### Effect of strawberry extracts pretreatment on lipid peroxidation

**Figure 5:**
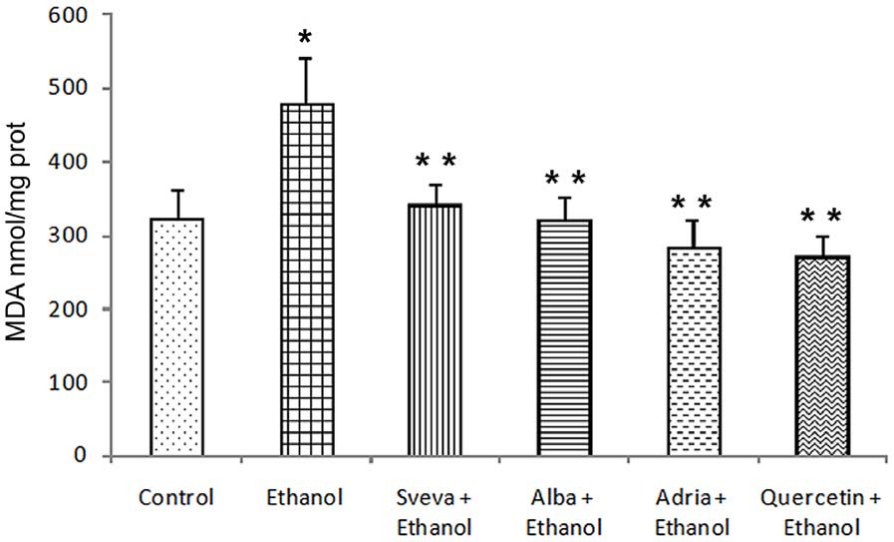
Effect of intragastric pretreatment with the different strawberry extract and 100 mg/kg of quercetin on the malondialdehyde concentration (nmol/mg prot.) in gastric mucosa. ^*^ Statistical significance (*p<0.05*) of difference in MDA concentrations in non-pretreated rats exposed to absolute ethanol (Ethanol) as compared to the control animals (control). ^**^ Statistical significance (*p<0.05*) of difference in MDA concentrations in pretreated rats as compared to the non-pretreated rats exposed to ethanol (Ethanol).

Figure 5 shows the results of determination of MDA as index of lipid peroxidation in the gastric mucosa of rats exposed to ethanol and rats pretreated with strawberry extract or quercetin, as positive control. The MDA concentration was significantly increased (*p<0.05*) with the administration of ethanol compared to the control group (478.7±64.7 *vs* 323.1±38.4 nmol/mg protein, respectively). Pretreatment with strawberry extract applied i.g. significantly attenuated the MDA concentration in animals exposed to ethanol; in particular, MDA was significantly reduced (*p<0.05*) by treatment with the extracts of cultivars Adria, Alba and Sveva (285.6±36.4, 321.7±31.2 and 343.08±23.1 nmol/mg protein, respectively) when compared with the group exposed only to ethanol. Administration of quercetin prior to ethanol application also significantly restricted (*p<0.05*) the increase of MDA concentration. There were no significant differences among the groups pretreated with the different strawberry extracts and the gastroprotective effects were similar to those reported for quercetin.

### SOD and Cat activity

**Figure 6:**
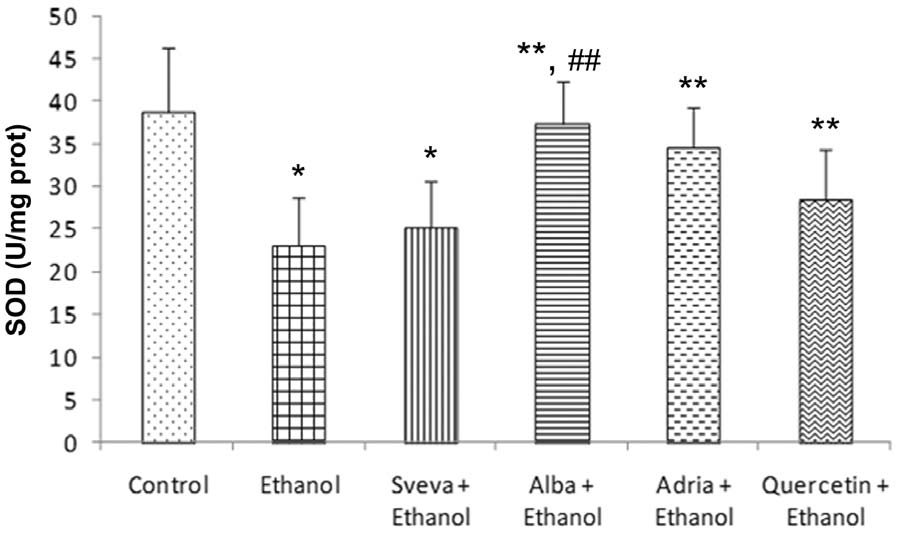
Effect of intragastric pretreatment with the different strawberry extract and quercetin on the superoxide dismutase activity (U/mg prot.) in gastric mucosa. ^*^ Statistical significance (*p<0.05*) of difference in SOD activity in non-pretreated rats exposed to ethanol (Ethanol) as compared to the control animals (Control). ^**^ Statistical significance (*p<0.05*) of difference in SOD activity in pretreated rats as compared to the non-pretreated rats exposed to absolute ethanol (Ethanol) group. ^##^ Significant difference in relation to the Sveva cultivar (*p<0.05*).

Regarding antioxidant enzymes, SOD activity was significant inhibited (*p<0.05*) in the gastric mucosa of animals exposed to ethanol (23.0±5.5 U/mg prot.), when compared to the respective value in intact gastric mucosa (38.7±7.4 U/mg prot.) (Control) (Fig. 6). On the contrary, pretreatment with Alba and Adria extracts applied i.g. reduced the decrease in SOD activity caused by ethanol; these effects were similar to those of quercetin and were statistically significantly (*p<0.05*) when compared with the group treated only with ethanol. The groups pretreated with Alba (37.2±5.0 U/mg protein) and Adria (34.5±4.6 U/mg prot.) showed the best results, highlighting significant differences (*p<0.05*) in relation to the group exposed only to ethanol and the group pretreated with Sveva extract.

**Figure 7:**
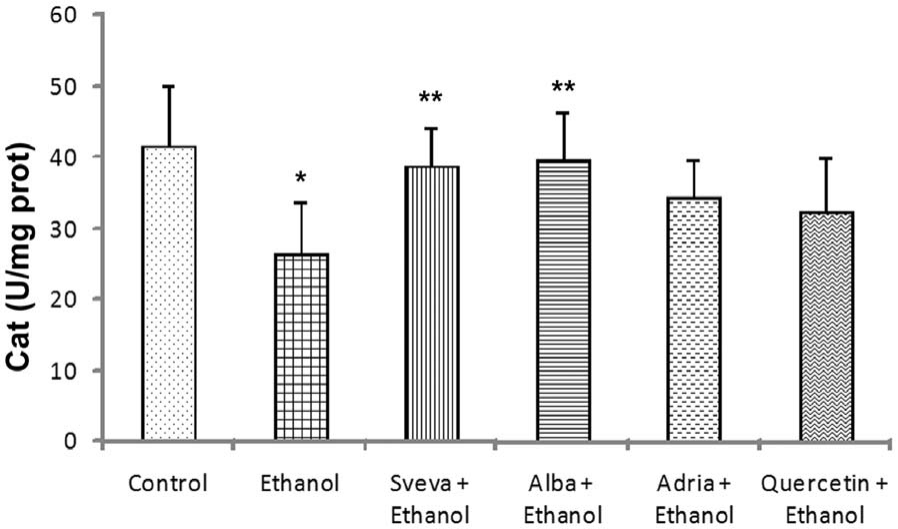
Effect of intragastric pretreatment with the different strawberry extract and 100 mg/kg of quercetin on the Cat activity (U/mg prot.) in gastric mucosa. ^*^ Statistical significance (*p<0.05*) of difference in Cat activity in non-pretreated rats exposed to ethanol (Ethanol) as compared to the control animals (Control). ^**^ Statistical significance (*p<0.05*) of difference in Cat activity in pretreated rats compared to the non-pretreated rats exposed to ethanol (Ethanol) group.

Moreover, Cat activity in gastric mucosa also significantly varied after ethanol, decreasing from 41.8±8.4 U/mg protein in normal gastric mucosa to 26.6±7.3 U/mg prot. in the group exposed to ethanol. Similarly to SOD, strawberry pretreatment significantly reduced the decrease of Cat activity (Fig. 7): in this case Alba cultivar showed the best results, differing significantly (*p<0.05*) from the other cultivars studied, as well as from controls and quercetin.

## Discussion

The antioxidant potential of fruit seems to play an important beneficial role in improving antioxidant defenses of the human body against the development of chronic diseases [31], [32].

It has been also demonstrated that substances with antioxidant properties, such as polyphenolic compounds, may protect against gastric-damaging effects caused by ethanol [10], [26]. The present study shows that anthocyanin-enriched strawberry extracts attenuate lesions in rat stomach caused by the i.g. application of noxious agents such as ethanol and that this protective effect is accompanied by the increase in Cat and SOD activities and reduction of MDA concentration, the most widely used index of lipid peroxidation, possibly related to the ability to scavenging oxygen free radicals by its polyphenolic constituents.

Previous studies documented that strawberries are important sources of phytochemicals. The major class of phenolic compounds in these berries is represented by flavonoids, followed by hydrolyzable tannins (ellagitannins and gallotannins), and phenolic acids (hydroxybenzoic acids and hydroxycinnamic acids) together with condensed tannins (proanthocyanidins), the latter being the minor constituents. Anthocyanins in the strawberry are the best known polyphenolic compounds and quantitatively the most important [7]–[9], so that more than 25 different anthocyanin pigments have been described from different varieties and selections [8]. In this study 9 anthocyanin pigments were identified, of which Pg derivatives were predominant, followed by the Cy derivatives. These compounds have been previously described in strawberries by many authors [8], [21], [32], [33], placing anthocyanins, together with ascorbic acid, folates [14] and ellagitannins, as one of the most important compounds in strawberries being responsible for several beneficial actions for human health. Moreover, it was shown that the strawberry possesses high *in vitro* antioxidant capacity which is positively correlated with the content of polyphenolic compounds and, specifically, anthocyanins [34], [35]. The antioxidant capacity of anthocyanins may be one of their most significant biological properties [36], in fact, their individual contribution to the TAC in the three strawberry cultivars employed in this research was around 25–40% [21]. One of the approaches used to assess the antioxidant activity was to examine directly the free radical production and inhibition by using the highly sensitive EPR spectroscopy, which is able to detect directly the presence and the concentration of oxygen free radicals. Since hydroxyl radicals are very unstable, an exogenous spin trap, reacting with the free radical species, was used, thus generating more stable adducts with characteristic EPR profiles. In the present study, it was found that strawberry extracts of the three cultivars were able to reduce the signal intensity of EPR, suggesting that they are very effective to scavenge these species competing with the spin trap for OH^•^. The evaluation of the antioxidant capacity of strawberry contributes to obtaining an overall picture of its capacity and potential to reduce oxidative reactions that can cause adverse health effects, even chronic diseases and cancers [37]. In this study, gastric mucosal injury was induced by administration of ethanol through intubation into rat stomach. The dose of the extract used in this *in vivo* study was based on the results reported by our group from a clinical study in which 500 g of strawberry daily significantly increased plasma TAC and ascorbic acid concentrations [14] as well as erythrocyte resistance to oxidative hemolysis [15]. It was observed that 40 mg of strawberry extract per kilogram of body weight had nearly the same antiulcerative activities as quercetin (Fig. 4), with the best results from the Adria extract. The administration of total anthocyanins from the crude extract was approximately 25 µg/kg of body weight for Adria and Sveva cultivar, while for Alba cultivar it was about 50 µg/kg. It has been previously reported [38], [39] that the mechanism of ethanol-induced gastric lesions is varied, including damaged mucosal blood flow and mucosal cell injury. In addition, ethanol-induced gastric mucosal damage is associated with overproduction of free radicals, which lead to an increased lipid peroxidation. Several investigations have reported the gastroprotective effect of different compounds found in strawberry, such as vitamin C and flavonoids, especially quercetin. It was found that addition of vitamin C significantly attenuated and reversed the aspirin-induced gastric damage in humans [40], [41]. The antiulcerogenic and anti-Helicobacter pylori activities of flavonoids from different plant extracts have been also reported [42], [43]. Moreover, it seems likely that one of the mechanisms by which the strawberry extract suppresses the development of gastric mucosal damage is the scavenging of active oxygen by its red pigment fraction. The high radical scavenging activity of anthocyanin is well known; in fact, the high correlation found between total anthocyanin content and % of UI inhibition suggests that these compounds can be related with the protective effects of the strawberry. Despite the fact that individual antiulcerative activities of each component of red pigment were not determined, it can be suggested that nearly all of the antiulcerative activities of strawberry extract could be attributed to its red pigment fraction composed of anthocyanin derivatives, as previously demonstrated in other berries [10]. In these studies, it was found that both black chokeberry extract and its hydrolysate administered at 2 g/kg of body weight had nearly the same protective effect as quercetin administered at 100 mg/kg of body weight in suppressing the area of gastric mucosal damage caused by the subsequent application of ethanol, to <30% compared to the control group. These gastroprotective effects were attributed, in part, to the increased mucus secretion of the stomach wall. Moreover, within the group of flavonoids, anthocyanosides are thought to act by influencing the biosynthesis of the mucopolysaccharides, thus improving the efficiency of the mucus barrier at the gastric level. Oral pretreatment with cyanidin 3-glucoside significantly inhibited the formation of ethanol-induced gastric lesions and the elevation of lipid peroxide levels. In addition, pretreatment with cyanidin 3-glucoside significantly increased the level of glutathione and activities of radical scavenging enzymes, such as superoxide dismutase, catalase, and glutathione peroxidase, in gastric tissue [44]. Other mechanisms have been proposed to explain the gastroprotective effect of flavonoids; these include the increase of mucosal prostaglandin content [45] and the decrease of histamine secretion from mast cells by inhibition of histidine decarboxylase [46]. In addition, flavonoids have been found to have a powerful free radical scavenger activity [37] that could play an important role in ulcerative and erosive lesions of the gastrointestinal tract. In the present study, quercetin was used as a positive control. The capacity of quercetin to prevent gastric mucosal lesions produced by ethanol, or acidified ethanol, has been reported thereby increasing the amount of neutral glycoproteins in the gastric mucosa [47]–[49]. Since these proteins are the most abundant and possibly the most important in the gastric mucosa [50], it can be assumed that their quantitative replacement may represent a kind of “return” to normality of the mucosa, and thus a recovery of the defensive capacity against aggression produced by ethanol.

The involvement of strawberry extracts in the mechanism of gastric mucosal defense against the formation of gastric lesions caused by noxious substances has not received sufficient scientific attention. Here, we provide evidence that strawberry extracts applied topically caused a decrease of acute gastric lesions induced by ethanol. Previous studies have demonstrated that the damaging action of ethanol could be attributed to ROS enhancement, to lipid peroxidation and to inhibition of antioxidant enzyme activity caused by ROS [10], [26]. We found that strawberry extracts attenuated the rise in MDA content in the gastric mucosa injured by ethanol, thus indicating that the extract can attenuate the process of lipid peroxidation implicated in the pathogenesis of ethanol gastric damage; furthermore the suppression of the antioxidant enzymes was not observed. Moreover, strawberry extracts do not contribute to the suppression of gastric acid secretion which has been reported as one of the antiulcerative mechanisms [51]. The effect of strawberry intake may go further beyond the direct antioxidant effect. Data from *in vitro* experiments recently revealed that the antioxidant properties of strawberry flavonoids could lie in their localization in lipoprotein domains and cell membranes, which generally serve as targets for lipid peroxidation, suggesting a protective interaction of flavonoids with lipid bilayers [17]. This mechanism of action could partly explain the *in vivo* role for strawberry in mitigating fed-state oxidative stressors [18].

The important cellular antioxidant enzymes SOD and Cat, contribute to the gastric oxidative/antioxidative balance. A decrease of both SOD and Cat activities in gastric mucosa of rats exposed to ethanol leads to the accumulation of ROS and consequently to an increase in MDA concentration. In our investigation, ethanol induced inhibition of SOD and Cat activities, suggesting an important role for these enzymes in the pathogenesis of gastric injury. Other authors have reported results supporting this finding: SOD and Cat activities in rat stomach tissue decreased by indomethacin and HCl/ethanol-induced oxidative gastric mucosal damage [48]–[52], they also decreased in cold restraint stress [53], and gastric mucosal SOD activity significantly decreased after topical application of absolute ethanol [26], [51]. It is important to note that these enzymes have a bimodal behavior. In the short term, as in this case, it decreases after damage, while in the long term it increases; in both cases this may be an indicator of increased oxidative damage. The tendency to increase and subsequently recover the basal level of these enzymes might be interpreted as the consequence of the initial alteration of cellular steady-state function followed by induction phenomena tending to reestablish the primitive situation [54]–[56]. In this case, the short space of time between the induction of damage by ethanol and sacrifice makes it virtually impossible for an induction of the enzyme system; therefore, it is clear that in this case, the lower enzyme activity represents more damage. Moreover, Brzozowski et al. [51] demonstrated that ethanol decreased the gene expression and the activity of SOD in the gastric mucosa, suggesting that the suppression of key mucosal antioxidant enzyme, along with the elevation of lipid peroxidation, play an important role in the pathogenesis of these lesions. The increase, at mucosal level, of lipid peroxidation as well as the decrease in SOD activity was attenuated by strawberry extracts, suggesting that the reduction in lipid peroxidation may contribute to the attenuation of the deleterious effect of noxious agents on the gastric mucosa. These protective effects can be ascribed also to the fact that anthocyanin can elevate the gastric PGE_2_ production in animals treated with the extracts, as previously indicated [51], and in accordance with observations that some flavonoids stimulate PGE_2_ production by isolated gastric mucosal cells.

Based on our results and scientific literature, the potential of these extracts could also be considered for the prevention/treatment of chronic subacute injury at the stomach. In that sense, contemporary medications used in the treatment of gastric ulcers and other gastric-related pathologies involve the use of novel mucosal protective drugs. Several of these drugs based their action on antioxidative effects, and a number of recent studies are being conducted in the way to test the potential of some antioxidants as coenzyme Q_10_ [57] or vitamins C and E [58]. Even, anthocyanin-rich extracts from black rice have been already used for the treatment of gastric antral ulcerations in rats [59]. All these studies together plus results from the present study suggest that the intake of strawberry extracts during or after gastric pathological states might alleviate gastric mucosal damage, consequently preserving or attenuating from ulcer formation, side-effects of *H. pylori* treatment, subacute chronic alcohol intake or non-steroid anti-inflammatory-related gastritis.

The results here exposed demonstrated that strawberry extracts show an important gastroprotective effect against ethanol-induced gastric damage, probably related to their anthocyanin content and their ability to maintain the cell membrane integrity, reduce the free radical-dependent lipid peroxidation and preserve and/or activate endogenous antioxidant enzymes (SOD and Cat). All these features help to protect gastric mucosa from oxidative damage and to strengthen the mucosa barrier, the first line of defense against exogenous damaging agent. Certainly, the signaling pathways involved in the strawberry-induced activation of antioxidant enzymes against gastric injury are only beginning to be understood, and future studies should aim to test whether strawberry consumption affects antioxidant enzyme activity via modulation of gene expressions.
